# TEX15 is an essential executor of MIWI2-directed transposon DNA methylation and silencing

**DOI:** 10.1038/s41467-020-17372-5

**Published:** 2020-07-27

**Authors:** Theresa Schöpp, Ansgar Zoch, Rebecca V. Berrens, Tania Auchynnikava, Yuka Kabayama, Lina Vasiliauskaitė, Juri Rappsilber, Robin C. Allshire, Dónal O’Carroll

**Affiliations:** 1 Centre for Regenerative Medicine, Institute for Stem Cell Research, School of Biological Sciences, University of Edinburgh, 5 Little France Drive, Edinburgh, EH16 4UU UK; 2 Wellcome Centre for Cell Biology, University of Edinburgh, Edinburgh, EH9 3BF UK; 3 Cancer Research UK Cambridge Institute, University of Cambridge, Li Ka Shing Centre, Robinson Way, Cambridge, CB2 0RE UK; 4 Institute of Biotechnology, Technische Universität Berlin, Berlin, Germany

**Keywords:** Spermatogenesis, Piwi RNAs

## Abstract

The PIWI protein MIWI2 and its associated PIWI-interacting RNAs (piRNAs) instruct DNA methylation of young active transposable elements (TEs) in the male germline. piRNAs are proposed to recruit MIWI2 to the transcriptionally active TE loci by base pairing to nascent transcripts, however the downstream mechanisms and effector proteins utilized by MIWI2 in directing de novo TE methylation remain incompletely understood. Here, we show that MIWI2 associates with TEX15 in foetal gonocytes. TEX15 is predominantly a nuclear protein that is not required for piRNA biogenesis but is essential for piRNA-directed TE de novo methylation and silencing. In summary, TEX15 is an essential executor of mammalian piRNA-directed DNA methylation.

## Abstract (web summary)

The PIWI protein MIWI2 counteracts transposon activity by transcriptional silencing in the mammalian germline. Here, the authors show that TEX15 interacts with MIWI2 and is required for piRNA-directed methylation of transposable elements in male germ cells.

## Introduction

The mammalian germline is derived from somatic cells during early development which necessitates the erasure and resetting of genomic DNA methylation patterns^1^. In the mouse male germline, the process of de novo DNA methylation occurs in foetal gonocytes during late gestation. Many young active long interspersed nuclear element-1 (LINE1) and intracisternal A-particle (IAP) copies escape the first round of de novo genome methylation^2^. These active TEs are silenced through post-transcriptional and transcriptional silencing mechanisms by PIWI proteins and their associated piRNAs^3^. The PIWI protein MILI (PIWIL2) initiates effector piRNA production through the piRNA-guided endonucleolytic cleavage and destruction of cytoplasmic TE transcripts^4,5^. Effector piRNAs are loaded into the PIWI protein MIWI2 (PIWIL4) that licence its entry to the nucleus and the ribonucleoprotein particle (RNP) is proposed to guide de novo methylation by tethering to nascent TE transcripts and the recruitment of effector proteins^4,6,7^. SPOCD1 was recently identified that links MIWI2 to the de novo methylation machinery^8^ but the full complement of MIWI2 effector proteins remains unknown. Here we show that TEX15 interacts with MIWI2 in foetal gonocytes and is required for piRNA-directed de novo DNA methylation of transposons.

## Results

### TEX15 interacts with MIWI2 in foetal gonocytes

**Fig. 1:**
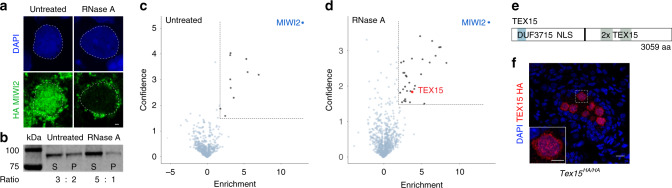
MIWI2 interacts with TEX15 in gonocytes undergoing de novo genome methylation. **a** HA stained *Miwi2*^*HA/+*^ E16.5 testes section untreated or treated with RNase A (*n* = 3). DNA stained with DAPI. Scale bar 1 µm. **b** Western blot of HA-MIWI2 from pellet (P) and soluble fraction (S) of *Miwi2*^*HA/HA*^ E16.5 testis lysates prepared with or without RNase A (*n* = 3). The ratio of HA-MIWI2 in the soluble and pellet fractions is shown, samples were processed in parallel and ratio between S and P acts as internal control. **c**, **d** Volcano plots showing enrichment (log_2_(mean LFQ ratio HA IP *Miwi2*^*HA/+*^ per control HA IP *Miwi2*^*+/+*^) over statistical confidence (−log_10_(*P* value of two-sided Student’s *t*-test)) of proteins co-immunoprecipitated with HA-MIWI2 from E16.5 testes lysates (**c**) untreated, (**d**) RNase A treated during protein extraction (*n* = 3). Highlighted proteins indicate >4-fold enrichment and significance *P* < 0.05. **e** Schematic representation of TEX15 domains and the nuclear localisation signal (NLS) prediction. **f** HA stained *Tex15*^*HA/HA*^ E16.5 testis section (*n* = 5). DNA stained with DAPI. Scale bar 10 µm. Bottom left panel shows close up of highlighted gonocyte. Scale bar 5 µm.

We hypothesised that the tethering of the MIWI2 RNP to the nascent transcript could be used to devise a strategy to enrich for proteins that are required for the execution of nuclear MIWI2 function. We performed immunofluorescence (IF) on thinly cut unfixed foetal testis cryosections. The width of the section is less than the diameter of a gonocyte so the cells are effectively sliced open and material can diffuse into the surrounding solution unless it is anchored through an interaction. The treatment of cryosections with RNase A prior to fixation dramatically reduced MIWI2’s nuclear staining in gonocytes (Fig. 1a). In addition, the inclusion of RNase A during extraction increased MIWI2’s solubilisation in foetal testis lysates (Fig. 1b). We performed immunoprecipitation coupled with quantitative mass spectrometry (IP-MS) of MIWI2 from E16.5 testes extracts with or without RNase A treatment using the fully functional *Miwi2*^*HA*^ allele that encodes an endogenously HA-epitope tagged MIWI2 protein^9^. The addition of RNase A greatly increased the number of MIWI2 interacting proteins (Fig. 1c, d, Supplementary Tables 1 and 2). Among the RNase A-dependent interactions TEX15 immediately struck our attention as a putative executor of nuclear MIWI2 function because it contains a nuclear localisation sequence (Fig. 1e), its expression is restricted to the male germline^10^ as well as being abundantly expressed in foetal gonocytes (Supplementary Fig. 1a); and most importantly *Tex15* deficiency in the mouse leads to the exact same phenotype observed in piRNA pathway or de novo methylation machinery mutants, namely sterility due to early meiotic arrest^11^. Mutations in the human TEX15 are also associated with male infertility^12–17^. Furthermore, the MIWI2-TEX15 interaction was confirmed from the analysis of an independent HA-MIWI2 IP-MS published dataset^8^ where the interaction is observed only in extracts prepared with Benzonase (Supplementary Fig. 1b), a nuclease that is commonly used to solubilise chromatin-bound proteins. *Tex15* encodes a large protein encompassing 3059 amino acids of unknown molecular function that contains a DUF3715 and two TEX15 domains (Fig. 1e). We generated a fully functional C-terminal HA epitope tagged *Tex15* (*Tex15*^*HA*^) allele (Supplementary Fig. 2a–c) and found that TEX15 expression in foetal testis is restricted to germ cells where it is predominantly localised to the nucleus (Fig. 1f).

### TEX15 is required for TE silencing in the male germline

**Fig. 2:**
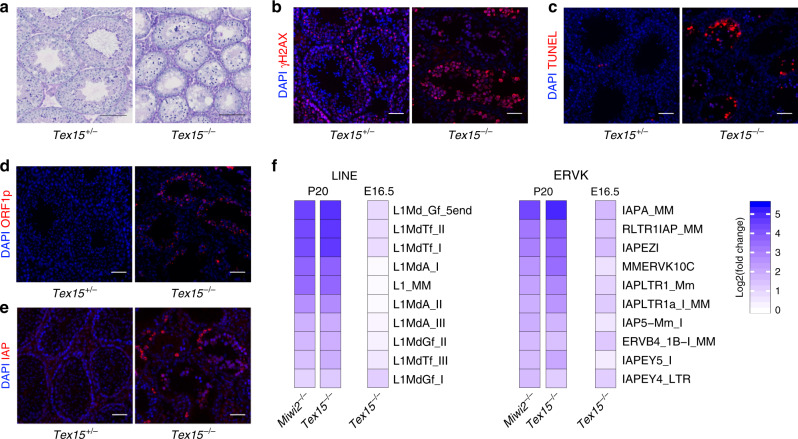
TEX15 is required for TE silencing. **a** Representative images of testis sections from adult *Tex15*^*+/−*^ and *Tex15*^*−/−*^ mice stained with PAS and Haematoxylin (*n* = 3). Scale bar 50 µm. **b**, **c** Representative images of testis sections of (*n* = 3) adult *Tex15*^*+/−*^ and *Tex15*^*−/−*^ mice stained for (**b**) γH2AX showing DNA damage response or (**c**) TUNEL visualising apoptotic cells. DNA stained with DAPI. Scale bar 50 µm. **d**, **e** Adult *Tex15*^*+/−*^ and *Tex15*^*−/−*^ testis sections (*n* = 3) stained for (**d**) LINE1 ORF1p or (**e**) IAP-GAG protein. DNA stained with DAPI. Scale bar 50 µm. **f** Heat map from P20 testes and E16.5 gonocytes RNA-seq as indicated showing fold change of the ten most upregulated LINEs and ERVKs in *Tex15*^*−/−*^ and *Miwi2*^*−/−*^ P20 testis compared to control (*n* = 3). *P* < 0.01, Benjamini–Hochberg adjusted two-sided Wald test.

The association of MIWI2 with TEX15, its nuclear localisation in foetal gonocytes and telling phenotype prompted us to explore if *Tex15* is required for TE silencing and de novo DNA methylation. We thus generated a *Tex15* null (*Tex15*^*−*^) allele in the mouse by CRISPR/Cas9-mediated genome editing of exon 5 that encodes the conserved DUF3715 domain (Supplementary Fig. 3a–d). The modified allele contains a 70 bp insertion in exon 5 that introduces in frame stop codons and should result in nonsense-mediated decay, and indeed a dramatic reduction of the *Tex15* transcript is observed in *Tex15*^*−/−*^ E16.5 foetal gonocytes (Supplementary Fig. 3e). In addition, the residual mutant transcript would encode a highly truncated TEX15 polypeptide encompassing the first 136 amino acids lacking any of its conserved domains. Most importantly, homozygosity of our *Tex15*^−^ allele fully recapitulated the published *Tex15*-deficent phenotype of male sterility, meiotic arrest coupled with extensive DNA damage and apoptosis^11^ (Fig. 2a–c, Supplementary Fig. 3f–i). TEX15 is required for both LINE1 and IAP silencing in the adult testis (Fig. 2d, e). To explore the full repertoire of TEs regulated by TEX15, we performed RNA-seq from post-natal day 20 (P20) mouse testes and found many TE families deregulated (Supplementary Fig. 4a, Supplementary Table 3). Importantly, we found that precisely the same families of TEs are deregulated in *Tex15*^*−/−*^ and *Miwi2*^*−/−*^ testes^8^ (Fig. 2f, Supplementary Fig. 4b). RNA-seq revealed that many of the TEs deregulated in P20 *Tex15*^*−/−*^ and *Miwi2*^*−/−*^ testes are also deregulated in *Tex15*^*−/−*^ E16.5 foetal gonocytes (Fig. 2f) which demonstrates a function for TEX15 in the foetal piRNA-pathway.

### TEX15 is not required for piRNA biogenesis

**Fig. 3:**
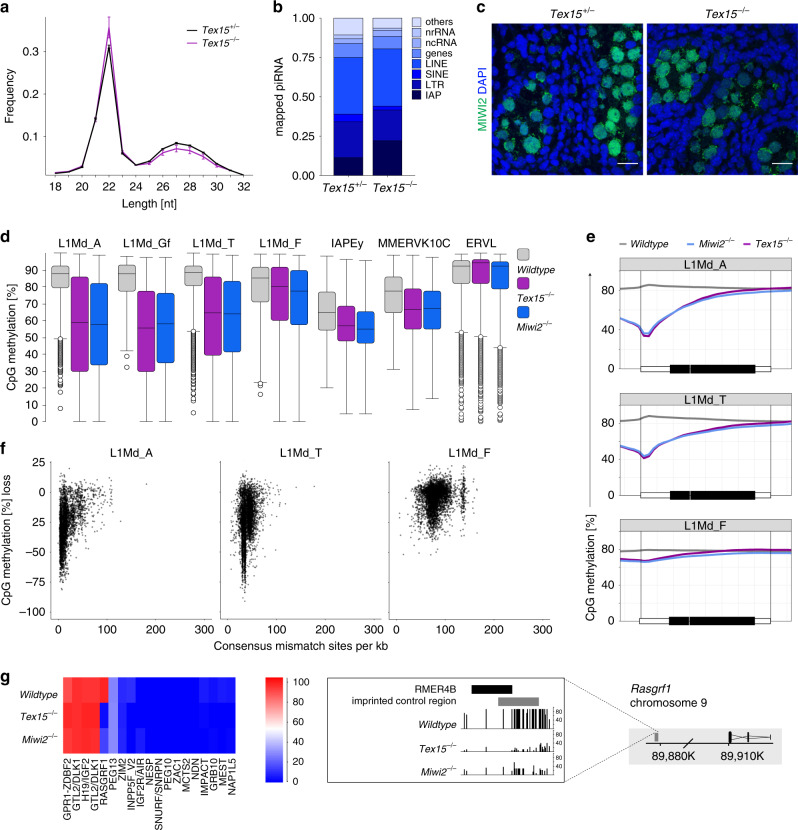
TEX15 is essential for piRNA-directed de novo genome methylation. **a**, **b** piRNAs analysis of *Tex15*^*+/−*^ and *Tex15*^*−/−*^ from small RNA sequenced E16.5 testes (*n* = 3). **a** Length distribution of small RNAs, data shows mean and s.e.m. *P* = 1, two sample *t*-test, Bonferroni adjustment. **b** piRNA annotation. **c** Representative images of MIWI2 stained E16.5 testis sections from *Tex15*^*+/−*^ and *Tex15*^*−/−*^ mice (*n* = 3). Scale bar 10 µm. **d**–**g** Genomic CpG methylation analysis from *Wildtype*, *Tex15*^*−/−*^ and *Miwi2*^*−/−*^ (*n* = 3) P14 undifferentiated spermatogonia. **d** CpG methylation percentage of TEs (non-overlapping genes). Box range indicates 25th to 75th percentile (interquantile range), line the median, whiskers range of median ± 2 interquantile range, dots datapoints outside this range. **e** Metaplots showing mean CpG methylation over LINE1 elements and adjacent 2 kb. **f** CpG methylation loss in *Tex15*^*−/−*^ relative to *Wildtype* plotted against divergence from consensus sequence. **g** Mean CpG methylation level of maternal and paternal imprinted regions, *Rasgrf1* shown in detail.

The dependency of young active TE silencing on TEX15 could indicate that TEX15 is required for execution of nuclear MIWI2 function or piRNA biogenesis amplification and loading; as the phenotypic outcome is identical in both scenarios. Sequencing small RNA from *Tex15*^*+/−*^ and *Tex15*^*−/−*^ E16.5 foetal testes did not reveal any major impact of *Tex15-*deficiency on small RNA length distribution (Fig. 3a), annotation of mapped piRNAs (Fig. 3b), piRNA amplification (Supplementary Fig. 4c, d) or piRNAs mapping to TEs (Supplementary Fig. 4e). The loss of piRNA biogenesis, amplification or loading results in the pronounced reduction of MIWI2’s nuclear localisation^4,9,18^. Thus, the normal localisation of MIWI2 in the absence of *Tex15* (Fig. 3c) corroborates the fact that TEX15 is not required for piRNA processing. RNA-seq from E16.5 foetal gonocytes excludes the possibility that TEX15 may function as a transcription factor required for gene expression, as a total of only 13 genes exhibited altered expression levels in the absence of *Tex15*. With the exception of *Tex15*, none of the subtly deregulated genes are associated with the de novo methylation or piRNA pathways (Supplementary Fig. 4f, Supplementary Table 4). In summary, these data do not support a role for TEX15 being a piRNA biogenesis nor a transcription factor.

### TEX15 is required for piRNA directed de novo DNA methylation

The demonstration that TEX15 interacts with MIWI2, localises to the nucleus and is not required for piRNA processing collectively indicates that TEX15 could be required for MIWI2-directed TE methylation. We therefore isolated genomic DNA from *Tex15*^*−/−*^ P14 spermatogonia and performed whole genome methylation sequencing (Methyl-seq) that we compared to *Wildtype* and *Miwi2*^*−/−*^ P14 spermatogonia methylomes^8^ generated using the same technique. As is the case for *Miwi2*-deficiency, no major changes in methylation of *Tex15*^*−/−*^ spermatogonia were observed in genic, intergenic, CpG island, promoter regions or a conglomerate of all genomic transposons (Supplementary Fig. 5a). However, the young LINE1 families regulated by the piRNA pathway represented by L1Md_A, L1Md_T or L1Md_Gf as well as IAPEy and MMERVK10C failed to be fully methylated in *Tex15*^*−/−*^ spermatogonia (Fig. 3d). Methylation specifically at TE promotor elements and in young TEs is a hallmark of piRNA-directed methylation^2,8,19^ and DNMT3C, which has a specialised function in germline de novo TE methylation^20,21^. Metaplot analysis revealed defective de novo methylation specifically at TE promotor elements in *Tex15*^*−/−*^ spermatogonia of young LINE1 families exemplified by L1Md_T and L1Md_Gf compared to the older L1Md_F; as observed in *Miwi2*^*−/−*^ spermatogonia (Fig. 3e, Supplementary Fig. 5b). Furthermore, the loss of methylation was especially evident in young elements within the respective families (Fig. 3f, Supplementary Fig. 5c). Finally, MIWI2 function is essential for the de novo methylation of a single imprinted locus, *Rasgrf1*^22^. Consistently, *Rasgrf1* methylation alone requires TEX15 function among imprinted loci (Fig. 3g).

## Discussion

A very recent study identified TEX15 as a regulator of TE silencing and connected TEX15 to MILI during spermatogenesis^23^. Here, we show that TEX15 interacts with MIWI2 in foetal gonocytes that are undergoing de novo DNA methylation thus directly link TEX15 to the process of piRNA-directed DNA methylation. Interestingly, the detection of the MIWI2–TEX15 interaction is dependent upon using either RNase A or Benzonase in the protein extraction procedure which likely indicates that the association is occurring in the context of chromatin. The localisation of MIWI2 to the nucleus coupled with normal piRNA biogenesis in the absence of TEX15 also clearly demonstrates that TEX15 is required for MIWI2’s nuclear function. Indeed, we unequivocally show that TEX15 is required for de novo DNA methylation of precisely the same TEs that are regulated by the MIWI2-piRNA pathway. Interestingly, TEX15 was not found to interact with SPOCD1 in foetal gonocytes^8^, which places a function for TEX15 upstream or in parallel to SPOCD1 in the recruitment of the de novo methylation machinery. The DUF3715 domain of TEX15 is found in two other proteins, TASOR (FAM208A) and TASOR2 (FAM208B), of which TASOR is a critical component of the HUSH complex that mediates TE silencing in somatic cells^24–26^. TASOR functions through the recruitment of MORC2 that stimulates the deposition of the repressive heterochromatin associated H3K9me3 mark^25,27^. MORC1 is an orthologue of MORC2 expressed in the developing male germline and essential for de novo methylation of young active TEs^28^. It is tempting to speculate that TEX15 may contribute to transcriptional TE silencing, possibly through H3K9me3. Indeed, transcriptional TE silencing is likely a prerequisite for de novo methylation as DNMT3L, a key component of the de novo methylation machinery, cannot interact with the transcriptionally active H3K4me3-modified chromatin^29^. In conclusion, we have identified TEX15 as an essential executor of piRNA-directed DNA methylation whose future study holds great promise in unravelling the molecular mechanisms underpinning this epigenetic event that is essential for the immortality of the germline.

## Methods

### Mouse strains and experimentation

The *Miwi2*^*HA*^ allele^9^ and *Miwi2*^*tdTomato*^ (*Miwi2*^*tdTom*^) allele^30^ have been previously generated in the O’Carroll laboratory and were kept on a C57BL/6N genetic background. Mouse alleles *Tex15*^−^ and *Tex15*^*HA*^ were generated using CRISPR/Cas9 gene editing technology^31,32^. For *Tex15*^*−*^ a single sgRNA (sgRNA-ex5b 5′-ACATCCATCACATCGGCCTG-3′) was injected together with CAS9 mRNA. For *Tex15*^*HA*^ a single sgRNA (sgRNA-C2 5′-AACAGAATCCGTTTCTACGA-3′) and a 194 bp single stranded DNA donor with 71 and 70 bp left and right homology arms respectively and a PAM site mutation (CTGCTTAGTATTAATAAACTTCAAATCTTTGTTTTGTTTTCTTCCAGCTCCATGGCAACAGAATCCGTTTCTACGAAGACCTGGAGGCGGAGGATCCTACCCATACGATGTTCCAGATTACGCTTAAAAATAAATCTCTTCATACTGAAATAAATGCAACTTAAGTTTTCTCAAGTATTTTTTGCCTACATGTT) were injected with Cas9 mRNA. Injections were performed on fertilised B6CBAF1/Crl (F_1_ progeny of cross between C57BL/6J and CBA/CaCrl) one-cell stage zygotes. F_0_ offspring for each line was screened for the desired allele by polymerase chain reaction (PCR) and Sanger sequencing. All lines descend from one founder animal, back crossed at least twice to a C57BL/6N genetic background resulting in a mixed B6CBAF1/Crl, C57BL/6N genetic background in the analysed mice. Primers used for screening and genotyping for *Tex15*^−^ mice were Tex15_ex5-116FW 5′-GACACAGCAGAGAACTGGAAGA-3′ and Tex15_ex5-116RV 5′-CCCAAGTATTCTCAGTGGGGAC-3′, for Tex15HA mice Tex15-C-HA-FW1 5′-TTTGCTGATGAATGGGCTTTGG-3′ and Tex15-C-HA-RV1 5′-ACAAGCCTCTTTATAACTGCATGG-3′. Genotyping of *Miwi2*^*HA*^ mice was done using the following primers: M2_ex1_Fw2 5′-ACAGCCACACCGTCTCTTTT-3′ and M2_int1-2_Rv 5′-CAGGATAGCCAAAGGAAGGA-3′, for *Miwi2*^*TdTom*^ mice Miwi2Tom-GenoF1 5′-TACTCCCAAACTCCGAGTCAC-3′, Miwi2Tom-GenoR1 5′-GTGCCTATCAGAAACGCAAGA-3′ and Miwi2Tom-GenoR2 5′-CTCCTAGCCAGAGTGCCTTTT-3′ were used. Foetal testes used in IP-MS were collected from *Miwi2*^*HA*^, back-crossed twice to an Hsd:ICR (CD1) outbred genetic background (*Miwi2*^*HA*^.CD1). Animals were mated and plugged females were separated from studs with the day of plug counted as E0.5. Testes for IP-MS experiments were collected at E16.5 from matings of *Miwi2*^*HA/HA*^ studs to *Miwi2*^*HA/HA*^ or Hsd:ICR (CD1) *Wildtype* females or from matings of Hsd:ICR (CD1) *Wildtype* studs to Hsd:ICR (CD1) *Wildtype* females. Male fertility was assessed by mating *Tex15*^*+/−*^ or *Tex15*^*−/−*^ studs to C57BL6/6N *Wildtype* females counting the number of embryos at E16.5. Animals were housed in open top (m3) or IVC-M cages with a 12 h light/12 h dark cycle at 19–23 °C and 30–70% humidity. Ethical approval for the mouse experimentation has been given by the University of Edinburgh’s Animal Welfare and Ethical Review Body and the work was done under license from the United Kingdom’s Home Office.

### Immunofluorescence

IF of HA-MIWI2 was performed on unfixed, freshly cut 8 µm cryo-sections of *Miwi2*^*HA/+*^ E16.5 foetal testes untreated or treated with 25 µg ml^−1^ RNase A (Sigma Aldrich) in phosphate-buffered saline (PBS) for 10 min at room temperature, followed by three 5 min washes with PBS and subsequent fixation with 3.7% formaldehyde in PBS for 10 min, followed by 2 washes with PBS and permeabilization in 0.1% Triton X-100 in PBS for 10 min. Sections were blocked for 30 min at room temperature in 10% normal donkey serum (Sigma), 1% bovine serum albumin (Sigma) and 0.1 M glycine (Sigma). Primary anti-MIWI2 (Ramesh Pillai lab, 1:200) was incubated overnight at 4 °C in blocking solution, donkey anti-rabbit Alexa Fluor 488 secondary antibody (Invitrogen) was used 1:1000 and incubated for 1 h at room temperature in blocking solution. DAPI (5 µg ml^−1^) was used as counter stain. Samples were mounted with Prolong Gold (Invitrogen).

IF using anti-MIWI2 (Ramesh Pillai lab, 1:500), anti-LINE1-ORF1p (O’Carroll lab, 1:500), anti-IAP-GAG (Bryan Cullen lab, 1:500) and anti-γH2AX (IHC-00059, Bethyl Laboratories, 1:500) was performed on freshly cut 6-µm sections of OCT embedded testes^9^. Fixation, permeabilization, blocking, antibody incubation and embedding as described above. DAPI (1 µg ml^−1^) was used as counter stain. Alexa Fluor (488, 568 or 647, Invitrogen) donkey anti-rabbit or donkey anti-mouse secondary antibodies (1:1000) were used in this study. Terminal deoxynucleotidyl transferase dUTP nick end labelling (TUNEL assay) was performed on paraformaldehyde (PFA) fixed paraffin embedded sections deparaffinized using 100% Xylene and a series of graded alcohols (descending 100–70%). Sections were treated with proteinase K (10 µg ml^−1^ in 10 mM Tris pH 8; Thermo Scientific) and labelled using Click-iT TUNEL assay, Alexa Fluor 647 dye (Invitrogen) according to the manufacturer’s instructions. Sections were then counterstained and embedded as described above.

IF of TEX15-HA from E16.5 foetal testis was performed on PFA fixed paraffin embedded tissues. Isolated E16.5 testes were rinsed with cold PBS and fixed in 2% PFA then transferred into 70% ethanol and paraffin embedded. Tissue sections of 4 µm were cut on a microtome (Leica), dried at 42 °C overnight and deparaffinized using 100% Xylene and a series of graded alcohols (descending 100–70%). For antigen retrieval sections were boiled in HIER solution (1 mM EDTA, 0.05% Tween-20 (Sigma) pH 8) and further treated with 0.4% Triton X-100 in PBS to permeabilize, then blocked for 1 h in blocking solution. Primary antibody (anti-HA (C29F4, Cell Signalling Technologies) 1:300), Alexa Fluor secondary antibody (donkey anti-rabbit 647 (Invitrogen) 1:1000), DAPI counterstain and mounting was done as described above.

Images were acquired on a Zeiss Observer (software Zen Blue), Leica SP8 confocal microscope (software Leica Application Suite X) or Zeiss LSM880 with Airyscan module (software Zen Black). If acquired using the Airyscan module, images were deconvoluted using Airyscan processing in the Zeiss Zen software set to 3D and recommended strength. Images were then processed and analysed with ImageJ (version 2.0.0-rc-65/1.51u).

### Western blotting

Snap frozen E16.5 *Miwi2*^*HA/HA*^ testes were homogenised using a micro-pestle and lysed for 10 min at room temperature in 100 mM KCl, 5 mM MgCl_2_, 0.5% Triton X-100 untreated or treated with RNase A (10 µg ml^−1^; Sigma Aldrich). The lysate was cleared for 10 min at 21,000 rcf, the supernatant taken as the soluble fraction and the pellet resuspended for 5 min at 95 °C with an equal volume of 2% sodium dodecyl sulfate (SDS), 50 mM Tris pH 8. Equal volumes of soluble and pellet fraction were then separated on 4–12% Bis–Tris Polyacrylamide gels (Invitrogen) according to the manufacturer’s instructions. Proteins were transferred onto 0.45 µm nitrocellulose membrane (Amersham, Protran XL), blocked in 3% skimmed milk and stained with primary antibody (anti-HA (C29F4 Cell Signalling Technologies) diluted 1:500 in blocking solution at 4 °C overnight, washed and incubated with Li-COR fluorescent conjugated secondary antibodies (anti-rabbit 800) diluted 1:10,000. Images were acquired and analysed using a Li-COR Odyssey imager. Ratio between soluble and pellet fraction was calculated from signal intensity of each band as measured by Image Studio Lite (version 5.2.5).

### Immuno-precipitation coupled mass-spectrometry (IP-MS)

E16.5 isolated testes were snap frozen in liquid nitrogen. A total of 50 testes per replicate were pooled, lysed and homogenised in 1 ml IP buffer (100 mM KCl, 5 mM MgCl_2_, 0.2% Tergitol NP-40) with complete protease inhibitor EDTA-free (Roche) without or with 25 µg ml^−1^ RNase A (Sigma) using 20 strokes in a glass douncer and incubated 30 min at 4 °C. Lysates were cleared for 5 min at 21,000 rcf and the supernatant incubated with 50 µl cross-linked (20 mM dimethyl-pimelidate in borate buffer pH 9) anti-HA magnetic beads (Pierce) for 30 min at 4 °C. Beads were washed four times in IP buffer followed by two flash washes on the magnet in KCless buffer (5 mM MgCl_2_, 0.1% Triton X-100) and eluted for 15 min at 50 °C in 100 µl 0.5% SDS, 50 mM Tris pH 8.0.

The eluted proteins were Trypsin digested as described^33^ peptides were desalted by STAGE tips^34^, then resuspended in 0.1% trifluoroacetic acid (v/v) and separated using ultra-high resolution nano-flow liquid chromatography nanoLC Ultimate 3000 fitted with an Easyspray (50 cm, 2 µm particles) column for peptide separation coupled to a high resolution/accurate-mass mass-spectrometer Orbitrap Fusion Lumos (Thermo Fisher Scientific). Non-linear gradient (2–40%–95% for 190 min) was used (mobile phase A 0.1% aqueous formic acid, mobile phase B 80% acetonitrile in 0.1% formic acid). MS was operated in data-dependent mode. MS acquisition parameters were the following: cycle time set to 3 s, MS1 scan was performed using Orbitrap resolution 120,000, RF lens 30%, AGC target 4.0e5, maximum injection time 50 ms, detected intensity threshold 5.0e3. MS2 scan was performed using Ion Trap in rapid scan setting with AGC target 2.0e4 and maximum injection time 50 ms. Raw data was processed with Max Quant (version 1.6.1.0) using Label-free quantitation (LFQ) IP-MS pipeline as described^35^ and peptides searched with standard settings against mouse UniProt database (July 2017)^36^. LFQ intensities were imported to Perseus^37^ (version 1.6.0.2) and processed as described^38^ for visualisation.

### Nuclear localisation signal (NLS) prediction

To predict an NLS the entire protein was searched for bipartite NLSs at a cut-off score of 5.0 with cNLS mapper^39^.

### Histology

Tissue was fixed in Bouin’s fixative overnight, washed in 70% ethanol and paraffin embedded. Sections measuring 4 µm were cut on a microtome (Leica) and deparaffinized using 100% Xylene and a series of graded alcohols (descending 100–70%). Periodic-acid-Schiff (PAS) staining was done using a PAS staining kit (TCS Biosciences) according to the manufacturer’s recommendations. Sections were de-hydrated using a reverse series of graded alcohols and Xylene, then mounted with Pertex mounting media (Pioneer Research Chemicals).

### Fluorescence activated cell sorting (FACS)

Gonocytes were FACS purified from control *Tex15*^*+/−*^; *Miwi2*^*tdTom/+*^ and experimental *Tex15*^*−/−*^; *Miwi2*^*tdTom/+*^ E16.5 testes using tdTomato expression as marker of gonocytes^8^. The isolated testes were dissected in a drop of Goni-MEM (DMEM (Life Techonologies) supplemented with penicillin-streptavidin (Life Technologies), NEAA (Life Technologies), sodium pyruvate (Life Technologies) and sodium lactate (Sigma-Aldrich)), transferred into 500 µl Trypsin-EDTA (0.25%, Gibco) and digested for 12 min at 37 °C, shaking. Digestion was stopped by the addition of 20% foetal calf serum (FCS) and 10 µl 5 mg ml^−1^ DNase I (Sigma-Aldrich), incubated 5 min at 37 °C and pelleted for 3 min at 200 rcf. The pellet was again treated with 10 µl DNase I (as above) and the cells resuspended in PBS with 2% FCS by pipetting at least 50 times. A 1 µg ml^−1^ DAPI was added to the cell suspension and tdTomato^+^ cells were sorted on a BD Fusion into PBS at 4 °C (Supplementary Fig. 6a) and snap frozen in liquid nitrogen.

For sorting of P14 CD9^+^ spermatogonia, testes were isolated, dealbulginated and digested with collagenase (0.5 mg ml^−1^, Sigma) for 10 min at 32 °C, shaking in 1 ml Goni-MEM. Seminiferous tubules were centrifuged at 200 rcf for 3 min and further digested in 1 ml Trypsin–EDTA (0.05%, Gibco) for 10 min at 32 °C, shaking. The digestion was stopped by adding 20% FCS. A total of 10 µl 5 mg ml^−1^ DNase I (Sigma-Aldrich) were added and further incubated for 3 min at 32 °C. Cells were blocked with Fc block (anti-CD16/32, clone 93, eBioscience, 1:50) and labelled with anti-CD45 (clone 30-F11, eBioscience, 1:200) and anti-CD51 (clone RMV-7, Biolegend, 1:50) biotin conjugated antibodies and stained with anti-CD9^APC^ (clone eBioKMC8, eBioscience, 1:200), anti-c-Kit^PE-Cy7^ (clone 2B8, eBioscience, 1:1600), streptavidin^V450^ (BD bioscience, 1:250) and 1 μg ml^−1^ DAPI. CD9^+^ cells were sorted into Goni-MEM at 4 °C on a BD Fusion or BD Aria II (Supplementary Fig. 6b) and pelleted for 5 min at 300 rcf and subsequently snap frozen in liquid nitrogen.

### RNA sequencing and analysis

For RNA-seq of FACS-isolated E16.5 gonocytes total RNA was extracted with QIAzol reagent (Qiagen) following the manufacturer’s recommendation. Libraries were prepared with RiboGone and the SMARTer Stranded RNA-seq kit for low input RNA-seq from Clontech and sequenced on a HiSeq 4000 (Illumina) in 75 bp paired-end mode. For RNA-seq from P20 testes total RNA was extracted from one testis with Qiagen RNeasy Mini kit (Qiagen) following the manufacturer’s protocol with the additional on-column DNase treatment. Libraries were prepared with NEBNext Ultra II Directional RNA Library Prep Kit for Illumina using eight PCR amplification cycles and sequenced on a NextSeq 500 (Illumina) in 150 bp singe-end read mode.

Reads were analysed for differentially expressed genes by mapping to GRCm38 genome_tran (release 84) with HISAT2^40^ using the options -no-mixed, -no-discordant, -qc-filter, -trim5 3 and counted with htseq-count^41^ (HTSeq 0.11.1) with the aid of GTF file. Differentially expressed genes were analysed using DESeq2^42^ (1.26.0). For the analysis of differentially expressed retrotransposons adaptor sequences were removed from the reads with cutadapt^43^ (1.8.1) using default settings. Consensus sequences of rodent retrotransposons were retrieved from Repbase^44^ (24.01) and used to map the processed reads using bowtie2^45^ (2.3.4.3) with default settings. Numbers of mapped reads per retrotransposon were counted and analysed using DESeq2^42^.

### Small RNA sequencing and analysis

Six foetal testes were used per replicate. RNA was isolated using QIAzol reagent following the manufacturer’s instructions. Size selection of 15–40 nucleotides (nt) from total RNA was performed using 15% TBE–Urea gel (Invitrogen) and a small RNA marker (Abnova R007) with 2× Gel Loading Buffer II (Ambion). To purify RNA from the gel, nuclease-free water was added to the cut gel slices, homogenised and incubated twice for 1 h at 37 °C, 1000 rpm with a freeze/thaw step on dry ice in between. Samples were transferred onto spin columns (Corning) plugged with filter paper (Whatman) and were centrifuged 1 min at max speed. RNA precipitation of the flow through was done with 2.5 volumes ethanol 100%, 1/10 volume 3 M NaOAc and 1 μl GlycoBlue (Life Technologies) overnight at −20 °C. RNA was washed in 80% ethanol and dissolved in 10 μl nuclease-free water. NEBnext Multiplex Small RNA Library Prep Set for Illumina (NEB) was used for library preparation following the manufacturer’s instructions with 4 μl size-selected RNA starting material, 1:2 diluted adaptors and 16 cycles PCR amplification. Qubit high sensitivity dsDNA kit on a Qubit fluorometer (Life Technologies) was used to measure the concentration and a HSD1000 tape on a Tapestation 2200 instrument (Agilent) to identify size and quality of the library. For the final library pool 4 ng were used per sample and sequenced on a HiSeq2500 sequencer (Illumina) in 50 bp single-end read mode. Adaptor sequences were removed from 3′ end of the raw fastq file with cutadapt^43^ using the options -m 16, -trimmed-only. Annotation of processed reads of 18–32 nt for each sample were retrieved as described^46^ using bowtie^45^ (1.2.1.1). Reads mapped to genomic TE sequences retrieved from RepeatMasker were allowed three mismatches. Mapped piRNA reads with 25–30 nt were categorised according to annotations, reads which did not map to any recorded genomic element are summarised in ‘other’. The piRNA amplification analysis and mapping of LINE1 and IAP was performed as described^4^. Consensus sequences of L1MdTfI and IAPEZI were retrieved from Repbase^44^ (24.01).

### Whole genome methylation sequencing (Methyl-seq) and analysis

For Methyl-seq DNA was isolated from FACS sorted P14 spermatogonial stem cells. Cells were digested using proteinase K 0.2 mg ml^−1^ in 10 mM Tris-HCl pH 8, 5 mM EDTA, 1% SDS, 0.3 M Na–acetate at 55 °C overnight, followed by two successive rounds of phenol/chloroform/isoamylalcohol (25:24:1, Sigma-Aldrich) extraction and one round of chloroform extraction. DNA precipitation was done by addition of 1/10 volume 3 M Na–acetate, 10 μg linear acrylamide (Invitrogen) and 1 volume of isopropanol and incubated at −20 °C overnight, washed two times with 70% ethanol and solubilized in 5 mM Tris-HCl pH 8. Libraries were prepared using the NEBnext Enzymatic Methyl-seq kit (NEB) according to the manufacturer’s protocol and sequenced on a HiSeq 4000 (Illumina) in 150 bp paired-end read mode. Analysis was performed as described in detail previously^8^. Genic regions were defined as probes overlapping genes and promoter as probes overlapping 2000 bp upstream of mRNA transcripts, as annotated by Ensembl (GRCm38.p6); CpG islands (CGIs) were defined as probes overlapping the Ensembl (GRCm38.p6) CGI annotation; reads overlapping transposons were removed for genic, promoters and CGIs genome. Transposon analysis was performed by unique mapping in the genome, excluding any repeats overlapping gene bodies. Transposon families were assessed by mapping to full length elements defined as follows: for LINE1 elements >5 kb; IAP families >6 kb; MMERVK10C > 4.5 kb. Intergenic regions were defined as regions which were non-overlapping with genes or transposons. Level of methylation was expressed as mean percentage of individual CG sites.

### Statistical information

Statistical testing was performed with R (3.3.1) using the R Studio software and with Perseus for the mass-spectrometry data. Unpaired, two-sided Student’s *t*-tests were used to compare differences between groups and adjusted for multiple testing using Bonferroni correction where indicated, except for RNA-seq data analysis where Wald tests and Benjamini–Hochberg correction were used. Averaged data are presented as mean ± s.e.m. (standard error of the mean), unless otherwise indicated. No statistical methods were used to predetermine sample size. The experiments were not randomised and the investigators were not blinded to allocation during experiments and outcome assessment.

### Reporting summary

Further information on research design is available in the Nature Research Reporting Summary linked to this article.

## Supplementary information

Supplementary Information

Reporting Summary

## Data Availability

The Methyl-seq data generated in this study have been deposited at ArrayExpress under the accession number E-MTAB-9090. The published Mehtyl-seq data of *Miwi2*^*−/−*^ and *Wildtype* spermatogonia was retrieved from E-MTAB-7997^8^. The sRNA-seq and RNA-seq data generated in this study have been deposited at Gene Expression Omnibus under the accession number GSE150350. Published RNA-seq data of P20 *Miwi2*^*−/−*^ testes was retrieved from GSE131377^8^. Data from the IP-MS experiments performed in this study have been deposited at ProteomeXchange under the accession number PXD019087. The published IP-MS experiments re-analysed in this manuscript were retrieved from ProteomeXchange PXD016701^8^. The Affymetrix microarray datasets for spermatogonia^30^ and spermatocytes^47^ and gonocytes^8^ were retrieved from ArrayExpress: E-MTAB-4828, E-MTAB-7067 and E-MTAB-7985, respectively. Full scans of the gels and blots are available in Supplementary Fig. 7. The source data underlying Figs. 1c, d, 2f, 3a, b, d–g and Supplementary Figs. 1a, 3e, f, h, 4a, c–f and 5a–c are provided in a Source Data file. All data are available from the corresponding author upon reasonable request. Source data are provided with this paper.

## Source data

Source Data

## Code availability

Scripts used for the Methyl-seq, RNA-seq and sRNA-seq analysis are available on github (https://github.com/rberrens/SPOCD1-piRNA_directed_DNA_met). Source data are provided with this paper.
