# Supplementary material for: TEX15 is an essential executor of MIWI2-directed transposon DNA methylation and silencing
Source: Nat Commun. 2020 Jul 27;11:3739. doi: 10.1038/s41467-020-17372-5 (PMC7385494; doi:10.1038/s41467-020-17372-5)
Supplement: Supplementary file 1 — Supplementary Information [file 41467_2020_17372_MOESM1_ESM.pdf]

Supplementary Information

TEX15 is an essential executor of MIWI2-directed transposon DNA methylation and silencing.

Schöpp *et al.*

This document contains:

- Supplementary Figures 1–7
- Supplementary Tables 1–4

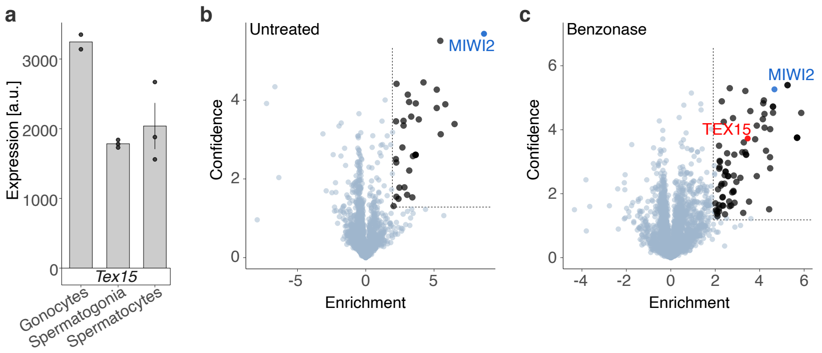

Supplementary Figure 1 | *Tex15* is abundantly expressed in foetal gonocytes and co-precipitates with HA-MIWI2.

a, Expression of *Tex15* in E16.5 gonocytes (n=2), adult spermatogonia (n=3) and Leptotene/ Zygotene spermatocytes (n=3) measured by Affymetrix microarray. Data shows mean of expression in arbitrary units (a.u.) and s.e.m. **b**, **c**, Volcano plots showing enrichment ($\log_2$(mean LFQ ratio of MIWI2-HA IP per control IP from *Miwi2*$^{+/+}$ foetal testis) over statistical confidence ($-\log_{10}$(*P*-value of two-sided Student's *t*-test)) of proteins co-immunoprecipitated with HA-MIWI2 from E16.5 testes lysates under hypotonic lysis conditions **b** without (n=3) and **c** with Benzonase treatment (n=4) respectively. The highlighted proteins indicate >4-fold enrichment and significance $P<0.05$.

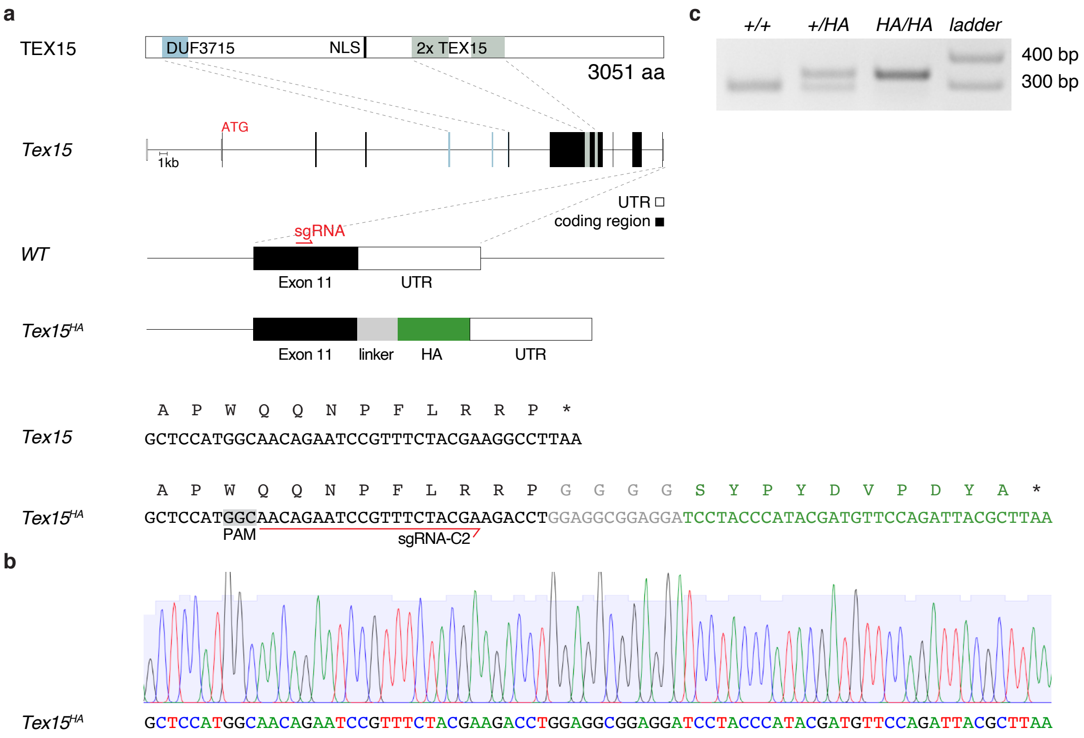

Supplementary Figure 2 | Generation of the *Tex15^{HA}* mouse allele.

a, Schematic representation of the TEX15 protein (transcript XM_006508977.3) and the *Tex15* locus with design of sgRNA, recognizing a specific sequence within exon 11 close to the protein's C-terminus. The 42 bp insertion encoding for a 4x glycine linker followed by HA epitope tag highlighted in grey and green respectively. **b**, Sequencing trace of *Tex15* allele carrying the HA epitope tag. **c**, Representative image of genotyping results for *Tex15$^{+/+}$*, *Tex15$^{HA/+}$* and *Tex15$^{HA/HA}$* animals.

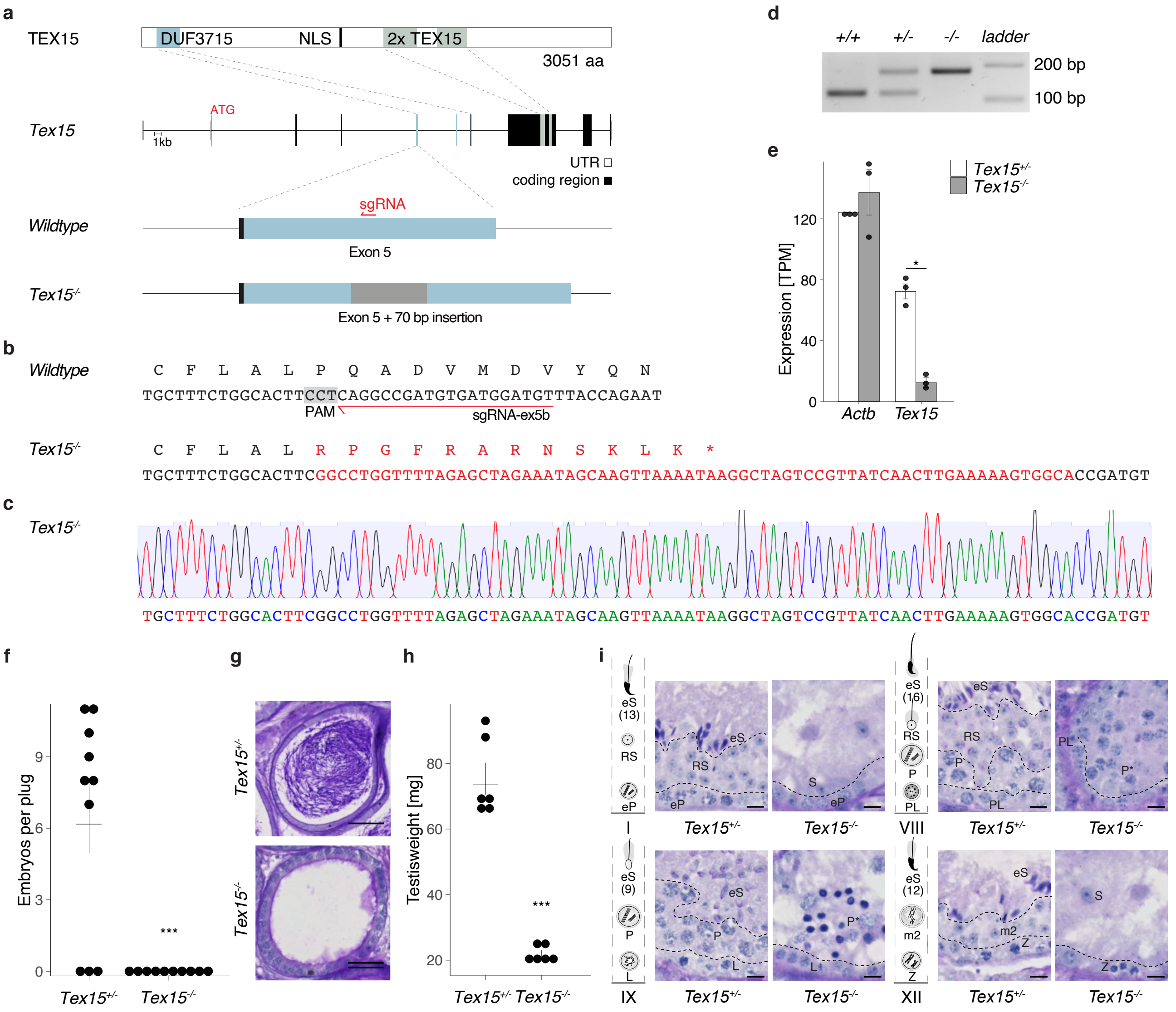

Supplementary Figure 3 | Generation and analysis of the *Tex15*⁻ allele.

a, Schematic representation of the TEX15 protein (transcript XM_006508977.3) and the *Tex15* locus and exon 5 with 70 bp insertion. **b**, Design of a sgRNA recognizing a specific sequence within DUF3715 leading to frame shift and a premature stop codon. The 70 bp insertion is highlighted in red. **c**, Sequencing trace of the mutated part of *Tex15*. **d**, Representative image of genotyping results for *Tex15*$^{+/+}$, *Tex15*$^{+/-}$ and *Tex15*$^{-/-}$ animals. **e**, RNA seq analysis of *Actb* and *Tex15* expression in transcripts per million (TPM) from E16.5 *Tex15*$^{+/-}$ and *Tex15*$^{-/-}$ gonocytes (n=3). Mean and s.e.m. indicated. *P<0.01, Bonferroni corrected two-sided Student's *t*-test. *Actb* P=0.85, *Tex15* P=0.001. **f**, E16.5 embryos per plug of *Wildtype* females mated with *Tex15*$^{+/-}$ (n=5) and *Tex15*$^{-/-}$ (n=4) studs respectively (10 plugs total for each genotype). Mean and s.e.m. indicated. ***, P<0.001, two-sided Student's *t*-test (P=0.00035). **g**, Representative images of epididymis section from adult *Tex15*$^{+/-}$ and *Tex15*$^{-/-}$ mice stained with PAS and Haematoxylin (n=3). Scale bar 10 μm. **h**, Testis weight from adult *Tex15*$^{+/-}$ and *Tex15*$^{-/-}$ mice measured in mg (n=3). Mean and s.e.m. indicated. ***, P<0.001, two-sided Student's *t*-test (P=8.283E-07). **i**, Detailed view of *Tex15*$^{+/-}$ and *Tex15*$^{-/-}$ adult testis section stained with PAS and Haematoxylin at different stages of spermatogenesis within seminiferous tubules. eP, early pachytene; RS, round spermatid; eS, elongated spermatid; S, Sertoli cell; P, pachytene; PL, preleptotene; L, leptotene; Z, zygotene; m2, diplotene (meiotic); P*, failing/dying pachytene cell. Scale bar 5 μm.

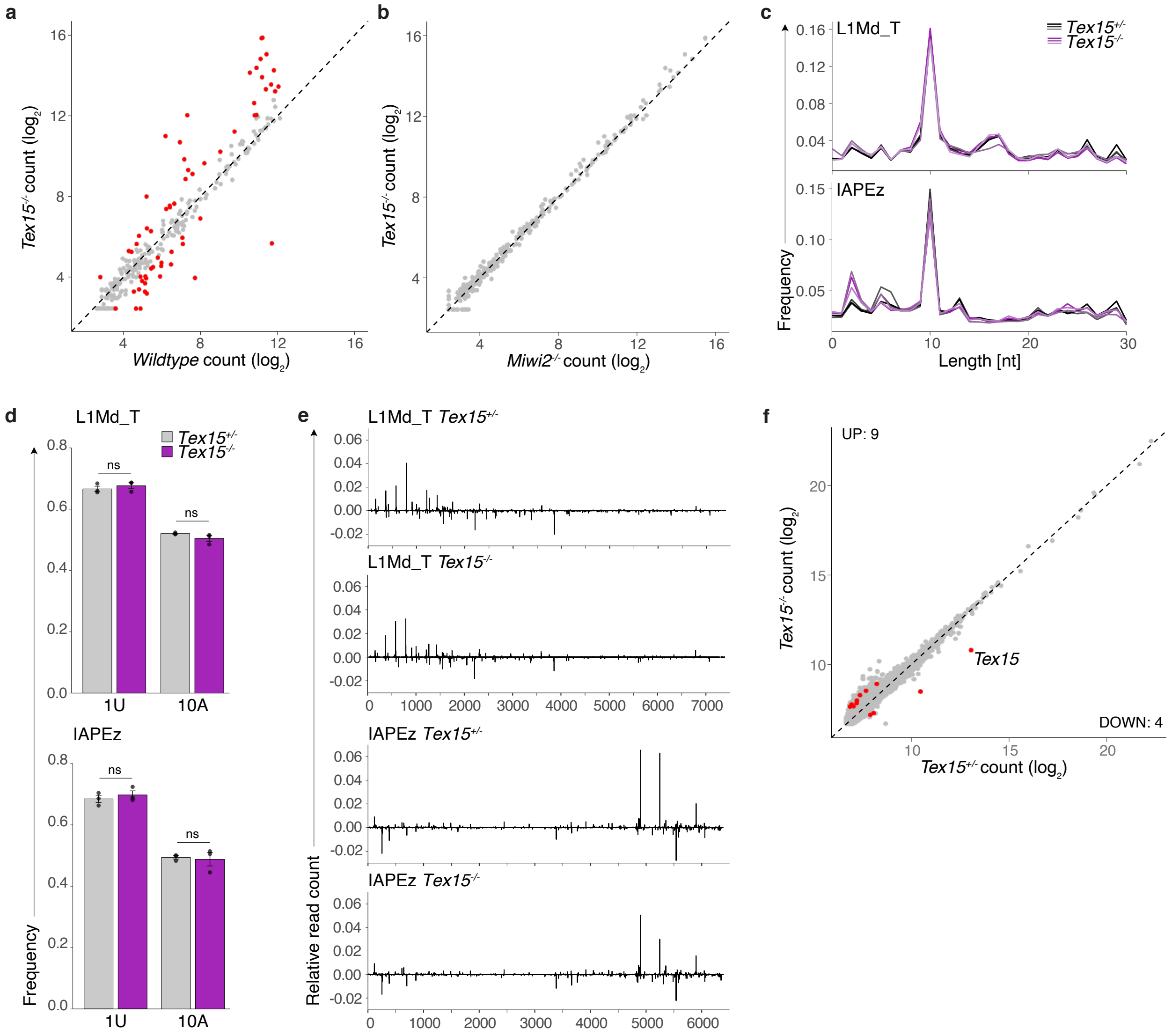

Supplementary Figure 4 I Deletion of *Tex15* causes TE upregulation but does not influence piRNA biogenesis.

a, **b**, Data acquired from RNA seq of (n=3) *Wildtype* and *Tex15*$^{-/-}$ P20 testes. **a**, Comparison of TE expression between *Wildtype* and *Tex15*$^{-/-}$ presented as read counts ($\log_2$). TEs with significantly different change in expression are highlighted in red. *P*<0.01, Benjamini-Hochberg adjusted two-sided Wald test, >2-fold change. **b**, Comparison of TE expression between *Tex15*$^{-/-}$ and *Miwi2*$^{-/-}$ mice presented as read counts ($\log_2$). **c**–**e**, Analysis of small RNA sequenced piRNAs from *Tex15*$^{+/-}$ and *Tex15*$^{-/-}$ E16.5 testes (n=3). **c**, Ping-pong analysis of piRNAs. Relative frequency of 5' end distance in nt between complementary piRNAs annotated to TE elements L1Md_T and IAPEz. **d**, piRNA nucleotide features. Relative amount of piRNAs annotated to TE elements L1Md_A and IAPEz with uracil at position 1 (1U) and adenine at position 10 (10A). Data represents mean (n=3) and s.e.m. ns, not significant *P*=1, two-sided *t*-test, Bonferroni adjusted. **e**, Relative read count of piRNAs annotated to L1Md_T and IAPEz, positive and negative values representing sense and antisense piRNAs respectively, plotted against position of consensus sequence. **f**, RNA seq analysis of gene expression in E16.5 *Tex15*$^{+/-}$ and *Tex15*$^{-/-}$ gonocytes (n=3), up- or down-regulated genes are highlighted in red. *P*<0.01, Benjamini-Hochberg adjusted two-sided Wald test, >2-fold change.

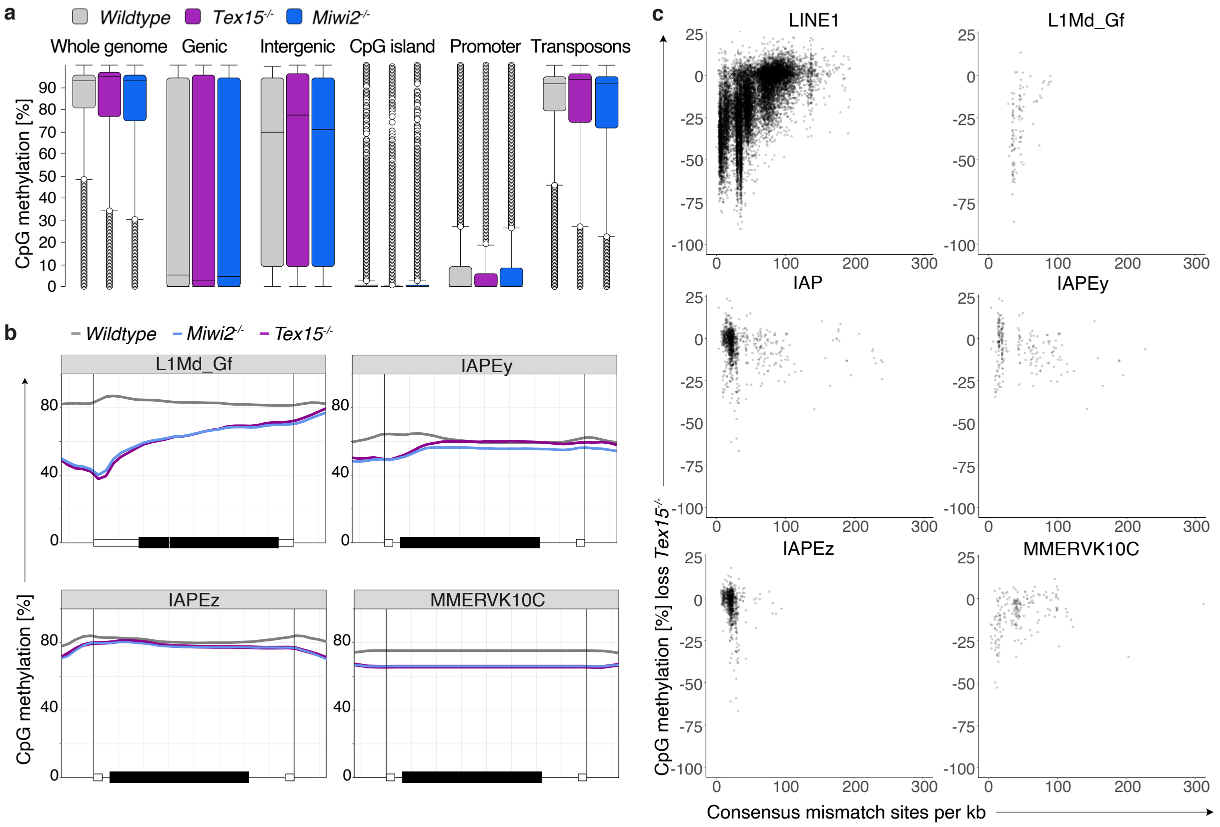

Supplementary Figure 5 | *Tex15* deficiency CpG methylation loss is restricted to young transposable elements.

a–**c**, Genomic CpG methylation analysis (P14 undifferentiated spermatogonia) in *Wildtype*, *Tex15*$^{-/-}$ and *Miwi2*$^{-/-}$ (n=3). **a**, CpG methylation percentage of genomic features genic, promoter and CpG islands and TEs (non-overlapping and intergenic non-overlapping TEs or genes). Box range indicates 25th to 75th percentile (interquantile range), line the median, whiskers range of median ±2 interquantile range, dots datapoints outside this range. **b**, Metaplots showing mean CpG methylation over L1Md_Gf, IAPEy, IAPEz, MMERVK10C and adjacent 2 kb. **c**, CpG methylation loss in *Tex15*$^{-/-}$ relative to *Wildtype* plotted against divergence from consensus sequence.

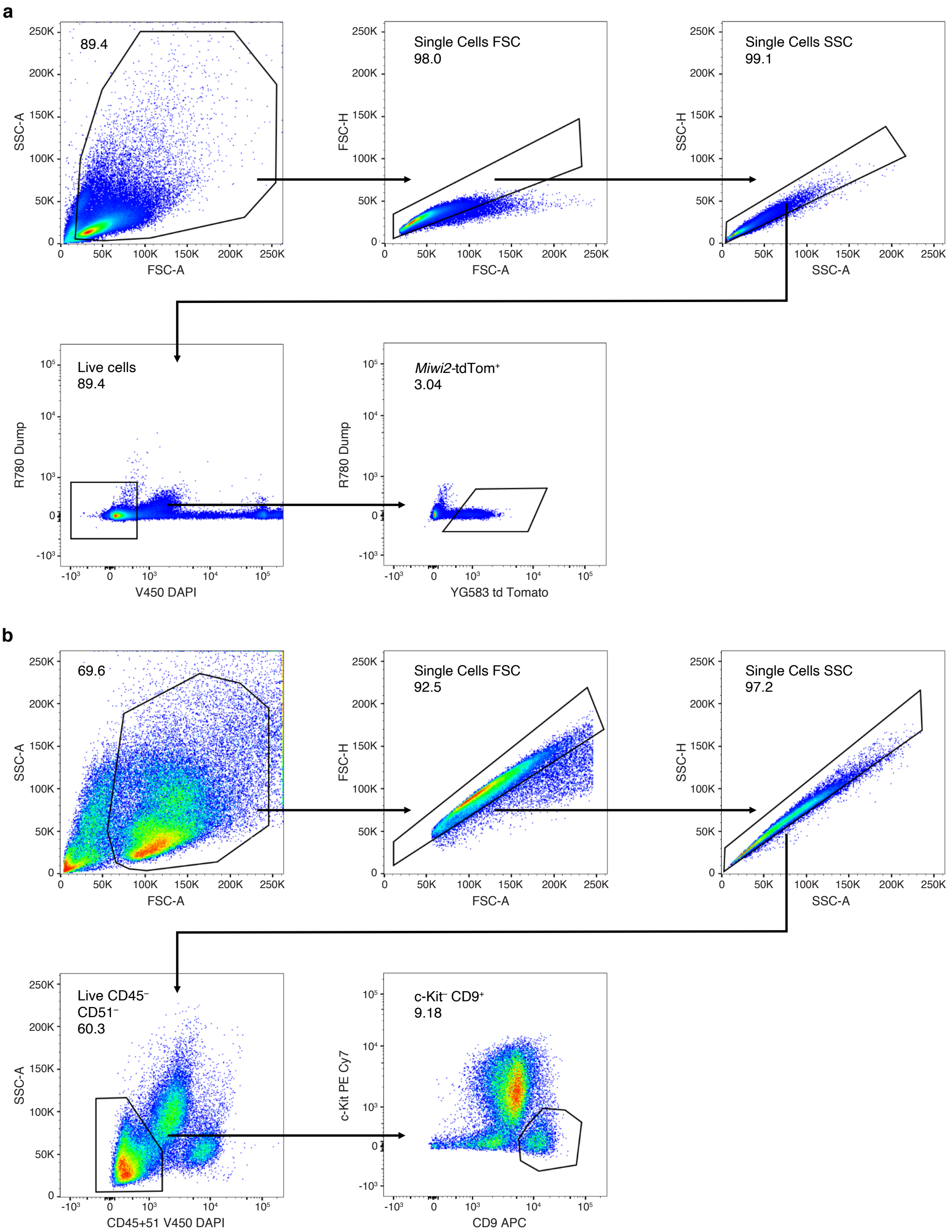

Supplementary Figure 6 | FACS sorting gating strategy.

a, Representative example of gating strategy for FACS isolation of E16.5 gonocytes using the *Miwi2*^{tdTOM} reporter allele. Live, *Miwi2*-tdTomato^{+} gonocytes from E16.5 testicular single-cell suspension was stained with DAPI. A representative experiment of n=3 mice is shown. **b**, Representative example of gating strategy for FACS isolation of P14 undifferentiated spermatogonia. Gates select live, CD45^{-} CD51^{-}, c-Kit^{-} CD9^{+} undifferentiated spermatogonia obtained from single-cell suspension of testicular cells stained with anti-CD45 biotin, anti-CD51 biotin, anti-c-Kit PE-Cy7 and anti-CD9 APC antibodies as well as streptavidin V450 and DAPI. Representative experiment of n=3 mice is shown.

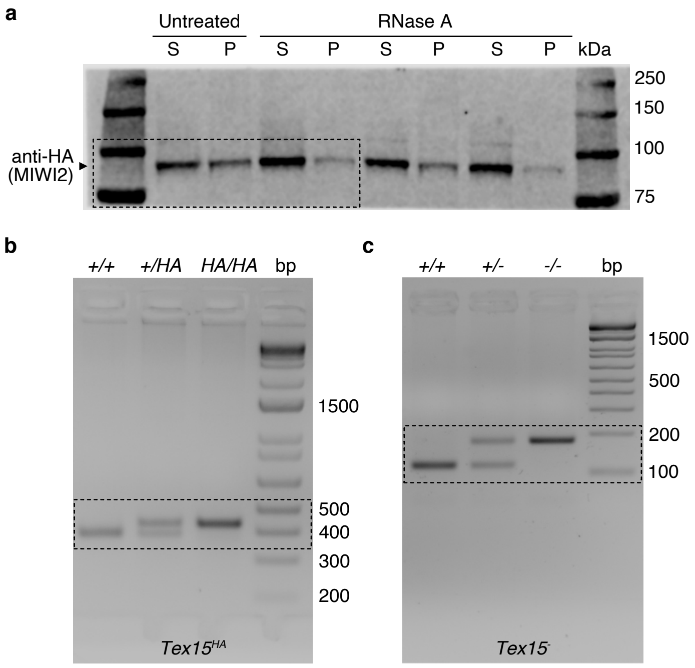

Supplementary Figure 7 | Uncropped original blot and gel scans.

a, Uncropped western blot image from Figure 1b. **b**, **c**, Uncropped gel images from **b** Supplementary Figure 2c and **c** Supplementary Figure 3d.

**Protein.ID**	**Protein**	**Gene**	**pvalue**	**Enrichment**
Q8CGT6	MIWI2	*Piwil4*	6.1E-06	7447.0
Q91XW8	FKBP6	*Fkbp6*	6.4E-04	122.3
D3YUE6	GTSF1	*Gtsf1*	5.1E-04	43.8
Q3U7K7	TRIM21	*Trim21*	1.6E-04	38.7
Q5SUE7	ADAD1	*Adad1*	5.0E-03	12.0
Q8CAE2	HENMT1	*Henmt1*	2.1E-03	9.0
Q5U4C1	GASP1	*Gprasp1*	9.3E-05	8.9
Q5VCS6	TDRD5	*Tdrd5*	9.6E-04	8.6
D3YWG8	MOV10L1	*Mov10l1*	1.1E-04	8.6
Q14BI7	TDRD9	*Tdrd9*	2.5E-02	5.4

Supplementary Table 1 | Proteins identified as MIWI2 interactors without RNase treatment

Table lists all statistically significant identified proteins with at least 4-fold enrichment in the HA-MIWI2 immunoprecipitation without RNase A treatment. $P<0.05$, two-sided Student's *t*-test.

Protein.ID	Protein	Gene	pvalue	Enrichment
Q8CGT6	MIWI2	*Piwil4*	1.6E-04	14532.5
D3YUE6	GTSF1	*Gtsf1*	2.3E-03	306.0
Q5SUE7	ADAD1	*Adad1*	8.3E-04	158.6
Q5VCS6	TDRD5	*Tdrd5*	1.4E-03	114.6
Q91XW8	FKBP6	*Fkbp6*	5.5E-04	84.1
Q14BI7	TDRD9	*Tdrd9*	2.2E-03	67.2
Q80Y52	HSP90AA1	*Hsp90aa1*	3.2E-02	41.5
Q61699	HSPH1	*Hsph1*	1.8E-03	35.1
Q3TJG6	PTGES3	*Ptges3*	4.7E-03	30.6
Q5U4C1	GASP1	*Gprasp1*	4.0E-04	28.0
Q3U7K7	TRIM21	*Trim21*	9.9E-03	17.6
Q8BVN9	MAELSTROM	*Mael*	1.2E-02	16.4
F8VPN2	TEX15	*Tex15*	1.5E-02	13.7
Q99MV7	RNF17	*Rnf17*	1.7E-03	13.3
Q3TX38	VDAC3	*Vdac3*	1.4E-02	11.7
Q9Z2X1	HNRNPF	*Hnrnpf*	2.9E-02	11.1
Q8CAE2	HENMT1	*Henmt1*	8.1E-03	10.5
Q4FJN2	FKBP5	*Fkbp5*	1.4E-02	8.5
P38647	HSP9	*Hsp9*	5.5E-03	8.2
Q3U1J4	DDB1	*Ddb1*	5.1E-03	7.7
Q8BP92	RETICULOCALBIN-2	*Rcb2*	2.7E-02	7.4
Q6P3E7	HDAC10	*Hdac10*	2.4E-02	7.2
Q3TXV1	PSMD2	*Psmd2*	1.0E-02	7.2
E9QNN1	DHX9	*Dhx9*	4.3E-03	6.8
Q8CDG1	MILI	*Piwil2*	2.6E-02	6.6
Q71LX8	HSP90AB1	*Hsp90ab1*	2.1E-03	6.3
B1ASB6	SPOCD1	*Spocd1*	4.3E-03	6.1
H3BKT1	C19orf84 homolog	*Gm38999*	1.2E-02	6.0
B2RQL4	ADAD2	*Adad2*	2.9E-02	5.5
P63017	HSPA8	*Hspa8*	2.2E-03	5.0
Q9EQI8	MRPL46	*Mrpl46*	1.6E-02	4.6

Supplementary Table 2 I Proteins identified as MIWI2 interactors upon RNase treatment
Table lists all statistically significant identified proteins with at least 4-fold enrichment in the HA-MIWI2 immunoprecipitation with RNase A treatment. $P<0.05$, two-sided Student's t-test.

TE	pvalue	Fold-Change
IAPA_MM	1.46E-156	5.041102846
L1Md_Gf_5end	1.17E-217	4.831110005
L1MdTf_II	<2E-324	4.719267306
L1MdTf_I	<2E-324	4.68459579
RLTR1IAP_MM	1.81E-157	3.89034751
L1MdA_I	3.55E-180	3.741926829
IAPEZI	1.04E-149	3.658763136
L1_MM	3.61E-158	3.621005321
MMERVK10C	2.72E-41	3.507031233
GSAT_MM	7.22E-25	3.292712845
IAPLTR1_Mm	7.68E-86	2.790532611
L1MdA_II	9.15E-102	2.758789976
IAPLTR1a_I_MM	1.59E-103	2.477258235
IAPEY5_I	6.76E-17	2.043293151
L1MdA_III	4.90E-58	1.958255321
L1MdTf_III	2.65E-05	1.916693679
L1MdGf_II	3.03E-53	1.879089312
IAP5-Mm_I	7.21E-31	1.732717725
SRV_MM-int	1.74E-07	1.618365494
IAPEYI	1.54E-18	1.607588348
RLTR46B	0.001239597	1.536346236
ERVB4_1-I_MM	4.05E-22	1.499804339
MMERGLN_I	1.03E-26	1.498855362
ERVB4_1B-I_MM	2.00E-12	1.465952283
MMERGLN_LTR	4.62E-07	1.452706061
MMVL30	1.06E-10	1.386165899
IAP5-Mm_LTR	0.001654352	1.318381681
RLTR25_MM	2.57E-12	1.299411918
ERVB4_3-I_MM	0.00592821	1.278398314
IAPLTR2_Mm	3.83E-10	1.257048972
ERVB4_1B-LTR_MM	6.04E-09	1.243593745
L1MdGf_I	4.49E-35	1.239962832
IAPEY4_I	1.70E-07	1.197267541
IAPEY4_LTR	5.10E-07	1.179967112
RLTR10B2	4.84E-06	1.088990604
ERV1-4_MM-LTR	0.000851454	1.048683221
L1MdA_IV	2.97E-18	1.019643936
B2_Mm1a	2.59E-14	1.018149183
ERVB4_2B-LTR_MM	0.002498859	-1.062756079
ERVB4_2-I_MM	8.23E-10	-1.145276136
RLTR13B2	7.83E-08	-1.248428089
RLTR19B_MM	0.001418549	-1.423584073
RLTR10D2	0.00101929	-1.429788906
RLTR13D2	1.50E-06	-1.500499825
RMER17B2	4.16E-06	-1.630125251
MERV1_I	1.06E-10	-1.656122187
RLTR43C	0.000701683	-1.795039358
RLTR9D	5.10E-07	-1.86330312
RMER2	0.000171046	-1.991702086
LTRIS5	0.00062669	-2.014260217
IAPEY3_LTR	7.78E-12	-2.379120854
RLTR50B	8.84E-05	-2.426436473
RLTR9D2	3.35E-09	-2.742050006
ERVB7_3-LTR_MM	0.000225873	-2.759801837
RLTR41A2	0.001666553	-2.821558008
RLTR1F_Mm	7.24E-06	-3.666559464
RLTR41B	4.23E-06	-4.180078225
IAPLTR3	5.73E-33	-4.830905124
RLTR30B_MM	5.88E-06	-6.208325728
IAPLTR3_I	1.20E-97	-6.322743634
RLTR9A2	2.40E-06	-6.553159691

Supplementary Table 3 | Deregulated TEs in *Tex15*$^{-/-}$ testes at P20

Table lists all statistically significantly deregulated TEs in *Tex15*$^{-/-}$ animals compared to *Wildtype* identified by RNA seq in P20 testes. *P*<0.01, Benjamini-Hochberg adjusted two-sided Wald test, >2-fold change.

Ensemble	Gene Name	pvalue	Fold-Change
Up-regulated			
ENSMUSG00000027435	Cd93	7.30E-06	4.359319993
ENSMUSG00000026712	Mrc1	2.22E-06	2.668664142
ENSMUSG00000050079	Rspry1	2.22E-06	1.189224869
ENSMUSG00000036617	Etl4	0.000139745	2.019792755
ENSMUSG00000055435	Maf	0.000139745	5.332570952
ENSMUSG00000070462	Tlnrd1	0.000139745	3.9182001
ENSMUSG00000074743	Thbd	0.000139745	2.808271472
ENSMUSG00000031245	Hmgn5	0.004880105	2.541229877
ENSMUSG00000039706	Ldb2	0.006580283	3.170161606
Down-regulated			
ENSMUSG00000009628	Tex15	6.88E-08	-2.338311102
ENSMUSG00000075015	Gm10801	2.22E-06	-2.491763953
ENSMUSG00000075014	Gm10800	2.98E-05	-2.674913904
ENSMUSG00000095280	Gm21738	0.000139745	-2.613879571

Supplementary Table 4 I Deregulated genes in *Tex15*$^{-/-}$ gonocytes at E16.5

Table lists all statistically significantly up- or down-regulated genes respectively in E16.5 *Tex15*$^{-/-}$ gonocytes compared to *Tex15*$^{+/-}$ control. $P<0.01$, Benjamini-Hochberg adjusted two-sided Wald test, >2-fold change.
