# Supplementary material for: TEX15 is an essential executor of MIWI2-directed transposon DNA methylation and silencing
Source: Nat Commun. 2020 Jul 27;11:3739. doi: 10.1038/s41467-020-17372-5 (PMC7385494; doi:10.1038/s41467-020-17372-5)
Supplement: Supplementary file 2 — Reporting Summary [file 41467_2020_17372_MOESM2_ESM.pdf]

nature research

Corresponding author(s): Donal O'Carroll

Last updated by author(s): Jun 17, 2020

Reporting Summary

Nature Research wishes to improve the reproducibility of the work that we publish. This form provides structure for consistency and transparency in reporting. For further information on Nature Research policies, see our Editorial Policies and the Editorial Policy Checklist.

Statistics

For all statistical analyses, confirm that the following items are present in the figure legend, table legend, main text, or Methods section.

n/a	Confirmed	
☐	☒	The exact sample size (*n*) for each experimental group/condition, given as a discrete number and unit of measurement
☐	☒	A statement on whether measurements were taken from distinct samples or whether the same sample was measured repeatedly
☐	☒	The statistical test(s) used AND whether they are one- or two-sided<br>*Only common tests should be described solely by name; describe more complex techniques in the Methods section.*
☒	☐	A description of all covariates tested
☐	☒	A description of any assumptions or corrections, such as tests of normality and adjustment for multiple comparisons
☐	☒	A full description of the statistical parameters including central tendency (e.g. means) or other basic estimates (e.g. regression coefficient) AND variation (e.g. standard deviation) or associated estimates of uncertainty (e.g. confidence intervals)
☐	☒	For null hypothesis testing, the test statistic (e.g. *F*, *t*, *r*) with confidence intervals, effect sizes, degrees of freedom and *P* value noted<br>*Give P values as exact values whenever suitable.*
☒	☐	For Bayesian analysis, information on the choice of priors and Markov chain Monte Carlo settings
☒	☐	For hierarchical and complex designs, identification of the appropriate level for tests and full reporting of outcomes
☐	☒	Estimates of effect sizes (e.g. Cohen's *d*, Pearson's *r*), indicating how they were calculated

Our web collection on statistics for biologists contains articles on many of the points above.

Software and code

Policy information about availability of computer code

Data collection	Microscope images were acquire using the software Zeiss Zen (versions Blue and Black) as well as Leica Application Suite (LAS-X 3.5.5).<br>BD FACS Diva (8.0.1) was used for cell sorting.
Data analysis	The software used to analyse the data is described described previously (Zoch et al. 2020) as cited in the respective methods sections, the scripts used to analyse data and generate graphs has been made available at https://github.com/rberrens/SPOCD1-piRNA_directed_DNA_met. The following software was used:<br>bowtie 1.2.1.1<br>bowtie2 2.3.4.3<br>HISAT2 2.1.0<br>htseq-count 0.11.2<br>cutadapt 1.8.1<br>DESeq2 1.26.0<br>Repbase 24.01<br>R 3.3.1<br>Bismark v0.22.1<br>Trim Galore (v0.4.1,www.bioinformatics.babraham.ac.uk/projects/trim_galore/, Cutadapt65 version 1.8.1, parameters: --paired --length 25 --trim-n --clip_R2 5)<br>SeqMonk (www.bioinformatics.babraham.ac.uk/projects/seqmonk/)<br>Image Studio Lite 5.2.5<br>Zen black<br>Zen blue<br>Leica Application Suite X<br>Fiji ImageJ 2.0.0-rc-65/1.51u<br>Perseus 1.6.0.2

Max Quant 1.6.1.0
BD FACS Diva (8.0.1)
BD FloJo 8

For manuscripts utilizing custom algorithms or software that are central to the research but not yet described in published literature, software must be made available to editors and reviewers. We strongly encourage code deposition in a community repository (e.g. GitHub). See the Nature Research guidelines for submitting code & software for further information.

Data

Policy information about availability of data

All manuscripts must include a data availability statement. This statement should provide the following information, where applicable:

- Accession codes, unique identifiers, or web links for publicly available datasets
- A list of figures that have associated raw data
- A description of any restrictions on data availability

The Methyl-seq data generated in this study have been deposited at ArrayExpress under the accession number E-MTAB-9090 [http://www.ebi.ac.uk/arrayexpress/experiments/E-MTAB-9090].
The sRNA-seq and RNA-seq data generated in this study have been deposited at Gene Expression Omnibus (GEO) under the accession number GSE150350 [https://www.ncbi.nlm.nih.gov/geo/query/acc.cgi?acc=GSE150350].
Data for the IP-MS experiments were deposited at ProteomeXchange under the accession number PXD019087 [https://www.ebi.ac.uk/pride/archive/projects/PXD019087].
Data available under the accession numbers E-MTAB-7997, GSE131377 and PXD016701 were used for Data analysis of Methyl-seq, RNA-seq and IP-MS respectively.
Data available under the accession numbers E-MTAB-4828, E-MTAB-7067 and E-MTAB-7985 were used for Data analysis of Affymetrix microarray of spermatogonia, spermaocytes and gonocytes.
The following databases have been accessed: Uniprot (https://www.uniprot.org/, acessed July 2017), Repbase (version 24.01, https://www.girinst.org/server/RepBase/), UCSC repeat masker (https://genome.ucsc.edu/cgi-bin/hgTables, February 2019), Imprinted control regions from KCL atlas (https://atlas.genetics.kcl.ac.uk/, Schulz et al. 2008), Ensembl (GRCm38.p6, https://www.ensembl.org/Mus_musculus/).

Field-specific reporting

Please select the one below that is the best fit for your research. If you are not sure, read the appropriate sections before making your selection.

☒ Life sciences ☐ Behavioural & social sciences ☐ Ecological, evolutionary & environmental sciences

For a reference copy of the document with all sections, see nature.com/documents/nr-reporting-summary-flat.pdf

Life sciences study design

All studies must disclose on these points even when the disclosure is negative.

Sample size	Samples sizes were not determined by statistical methods, but by the amount of available biological material. For each experiment three or more biological replicates were analysed, to sufficiently calculate statistics.
Data exclusions	No datasets were excluded from the analysis.
Replication	All experimental data from mouse has been generated from a 3 biological replicates or as indicated, which showed consistent outcomes within each group for all experiments.
Randomization	Samples were grouped according to genotype. To control for covariates, control and experimental samples were always processed in parallel.
Blinding	No blinding was used during data acquisition and analysis due to the small number of samples processed at each time.

Reporting for specific materials, systems and methods

We require information from authors about some types of materials, experimental systems and methods used in many studies. Here, indicate whether each material, system or method listed is relevant to your study. If you are not sure if a list item applies to your research, read the appropriate section before selecting a response.

Materials & experimental systems

n/a	Involved in the study
☐	☒ Antibodies
☒	☐ Eukaryotic cell lines
☒	☐ Palaeontology and archaeology
☐	☒ Animals and other organisms
☒	☐ Human research participants
☒	☐ Clinical data
☒	☐ Dual use research of concern

Methods

n/a	Involved in the study
☒	☐ ChIP-seq
☐	☒ Flow cytometry
☒	☐ MRI-based neuroimaging

Antibodies

Antibodies used

anti-HA (C29F4, Cell Signaling Technologies, Lot#9, IF: 1:300 (TEX15-HA), 1:500 (HA-MIWI2));
anti-HA beads (88837, Pierce, Lot#SB246262 and Lot#UK285815, IP-MS: 50 µl)
anti-LINE1-ORF1p (di Giacomo et al. 2014, IF: 1:500);
anti-MIWI2 (a kind gift from R. Pillai, University of Geneva, CH, Reuter et al. 2013, IF: 1:500);
anti-IAP-GAG (a kind gift from B. Cullen, Duke University, Durham, NC, USA, Mietz et al. 1987 IF: 1:500);
anti-γH2AX (IHC-00059, Bethyl Laboratories, no Lot# available, IF: 1:500);
anti-CD16/32 (clone 93, eBioscience, Lot#E03559-1636, FACS: 1:50);
anti-CD45 (clone 30-F11, eBioscience, Lot#E02527-1635, FACS: 1:200);
anti-CD51 (clone RMV-7, Biolegend, Lot#Bl67752, FACS: 1:50);
anti-CD9 (clone eBioKMC8, eBioscience, FACS: 1:200);
anti-c-Kit (clone 2B8, eBioscience, Lot#E07591-1633, FACS: 1:1600);
anti-mouse (Alexa Fluor 488, 568, 647, Cat#A-21202, A10037, A32787, IF 1:1000)
anti-rabbit (Alexa Fluor 488, 568, 647, Cat#A-21206, A10042, A-31573, IF 1:1000)

Validation

The anti-HA antibody were validated for IF and in this study against mouse samples containing no HA epitope-tagged proteins the same has been done for WB previously (Zoch et al. 2020).
The anti-LINE1-ORF1p (described in di Giacomo et al. 2014), anti-MIWI2 (described in de Reuter et al. 2013) and anti-IAP-GAG (described in Mietz et al. 1987) antibodies have been previously validated for IF on mouse sections with and without the according proteins present and were used in several studies since.
The anti-γH2AX antibody has been previously used for IF on mouse sections (Vasiliauskaite et al. 2018, Much et al. 2016, Comazzetto et al. 2014) and yielded correct staining pattern characteristic for wildtype spermatocytes.
The anti-CD16/32, anti-CD45, anti-CD51, anti-CD9 and anti-c-Kit antibodies have been previously validated for FACS of undifferentiated spermatogonia (Vasiliauskaite et al. 2017).

Animals and other organisms

Policy information about studies involving animals; ARRIVE guidelines recommended for reporting animal research

Laboratory animals

Mice:
Miwi2-HA (B6CBAF1/Crl; C57BL/6N; Hsd:ICR (CD1)): male embryos E16.5
Miwi2-tdTomato (C57BL/6N): male embryos E16.5
Tex15-null (B6CBAF1/Crl; C57BL/6N): male embryos E16.5, males 14 days, males 20 days, males and females 8-16 weeks
Tex15-HA (B6CBAF1/Crl; C57BL/6N): male embryos E16.5
C57BL/6N: male embryos E16.5, males 8-16 weeks
Hsd:ICR (CD1): male embryos E16.5, males and females 8-16 weeks
B6CBAF1/Crl: fertilized 1 cell stage zygotes, males and females 8-16 weeks
B57BL/6J: females 8-16 weeks
CBA/CaCrl: males 8-16 weeks

Wild animals

The study involved no wild animals.

Field-collected samples

The study involved no samples collected from the field.

Ethics oversight

Ethical approval for the mouse experimentation has been given by the University of Edinburgh's Animal Welfare and Ethical Review Body and the work done under license from the United Kingdom's Home Office.

Note that full information on the approval of the study protocol must also be provided in the manuscript.

Flow Cytometry

Plots

Confirm that:

☒ The axis labels state the marker and fluorochrome used (e.g. CD4-FITC).

☒ The axis scales are clearly visible. Include numbers along axes only for bottom left plot of group (a 'group' is an analysis of identical markers).

☒ All plots are contour plots with outliers or pseudocolor plots.

☒ A numerical value for number of cells or percentage (with statistics) is provided.

Methodology

Sample preparation	The sample preparation has previously been described in detail (Visiliauskaite et al. 2017) as stated in the methods section. In brief, for sorting P14 spermatogonia testes were dealbuginated, digested at 32 ℃ in collagenase for 20 min, followed by 0.05 % Trypsin-EDTA for 10 min. Cells were further labeled with anti-CD16/32, anti-CD45, anti-CD51, anti-cKit and anti-CD9 antibodies. For sorting E16.5 gonocytes testes were digested in 0.25 % Trypsin-EDTA at 37 ℃ for 12 min.
Instrument	BD Aria II, BD Fusion
Software	BD FACS Diva (8.0.1), FloJo 8
Cell population abundance	P14 undifferentiated CD9+, c-Kit- spermatogonia abundance is ~3% of the live cell population. E16.5 Miwi2-tdTomato positive gonocyte abundance is ~2–3% of the live cell population.
Gating strategy	For P14 spermatogonia firstly, gates selecting the single cell population were set to encompass the population expressing the marker of interest. Cells were further gated for live and CD51/45 negative cells and sorted as CD9 high, c-Kit negative population as described previously (Vasiliauskaite et al. 2018). Gates were set based on fluorescence intensity in the full minus one (lacking the label for the marker of interest) and single stain samples for each label.<br>For E16.5 gonocytes firstly, gates selecting the single cell population were set to encompass the population expressing the marker of interest. Cells were further gated for live cells and sorted as tdTomato+ population as described previously (Vasiliauskaite et al. 2018). Gates were set based on fluorescence intensity compared to tdTomato- animals.

☒ Tick this box to confirm that a figure exemplifying the gating strategy is provided in the Supplementary Information.
